# Effect of electrical impedance-guided PEEP in reducing pulmonary complications after craniotomy: study protocol for a randomized controlled trial

**DOI:** 10.1186/s13063-022-06751-6

**Published:** 2022-10-01

**Authors:** Zihao Zhang, Lianqin Zhang, Jiang Zhu, Jun Dong, Hairui Liu

**Affiliations:** 1 Department of Anesthesiology, The Second Affiliated Hospital of Soochow University, 1055 Sanxiang Road, Suzhou, 215004 Jiangsu China; 2 Department of Neurosurgery, The Second Affiliated Hospital of Soochow University, 1055 Sanxiang Road, Suzhou, 215004 Jiangsu China

**Keywords:** Randomized controlled trial, Individualized PEEP, Pulmonary complications, Craniotomy

## Abstract

**Objective:**

The purpose of this study is to explore whether electrical impedance tomography (EIT)-guided individualized positive end-expiratory pressure (PEEP) can reduce the incidence of pulmonary complications within 1 week following a craniotomy compared with a single PEEP (PEEP = 6 cmH_2_O) from dura suturing to extubation.

**Methods:**

A randomized controlled trial will be conducted at the Second Affiliated Hospital of Soochou University. Five hundred forty patients undergoing a craniotomy in the supine position will be randomly allocated into the P6 (PEEP = 6 cmH_2_O) or Pi (individualized PEEP) group. Both groups of patients will receive a lung recruitment maneuver before suturing the dura. Then, the P6 group will receive 6 cmH_2_O PEEP, and the Pi group will receive EIT-guided individualized PEEP. The incidence and severity score of pulmonary complications within 1 week following surgery, the lung ultrasound score (LUS), regional cerebral oxygen saturation (rScO_2_), and PaO_2_/FiO_2_ before anesthesia (T0), 10 min after extubation (T1), 24 h after extubation (T2), and 72 h after extubation (T3) will be compared between the two groups. The duration of surgery and anesthesia, the level and duration of PEEP during surgery, the volume of liquid intake and output during surgery, and the postoperative ICU and hospital stays will be recorded. The main outcome of this study will be the incidence of pulmonary complications within 1 week after surgery.

**Discussion:**

The purposes of this study are to determine whether EIT-guided individualized PEEP from the beginning of dura suturing to extubation reduces the incidence of pulmonary complications within 1 week after a craniotomy compared with a single constant PEEP and to evaluate the length of ICU and hospital stays. If our results are positive, this study will show that EIT-guided individualized PEEP is better than a single constant PEEP and can further improve the prognosis of neurosurgical patients and reduce hospitalization costs, which will promote the wide application of individualized PEEP in clinical anesthesia.

**Trial registration:**

Chinese Clinical Trial Registry CHiCTR2100051200. Registered on 15 September 2021.

**Supplementary Information:**

The online version contains supplementary material available at 10.1186/s13063-022-06751-6.

## Introduction

Postoperative pulmonary complications (PPCs) are common, with an incidence of 39% [1–3]. Miskovic et al. [4] broadly described PPCs as “complications affecting the respiratory system after anesthesia and surgery,” while most studies describe PPCs as atelectasis, pneumonia, pulmonary edema, exacerbation of underlying chronic lung disease, or postoperative respiratory failure [5]. Patients undergoing a craniotomy are exposed to intraoperative mechanical ventilation, postoperative bed rest, limb paralysis, and other factors for a long time, thus resulting in obstruction of sputum drainage, which significantly increases the risk for PPCs [6, 7]. In addition, a postoperative state of low consciousness caused by postoperative hydrocephalus, posterior fossa edema, surgical site hematoma, brainstem infarction, and meningitis are independent predictors of PPCs in neurosurgical patients [8, 9]. PPCs have adverse impacts on prognosis, including an increased ICU occupancy rate, prolonged hospital stay, perioperative mortality, and increased hospitalization costs for non-cardiothoracic surgery patients [10–12].

Protective lung ventilation (PLV) strategies have been adopted by many anesthesiologists and are widely used in clinical anesthesia [13, 14]. A low tidal volume combined with positive end-expiratory pressure (PEEP) ventilation and an alveolar recruitment maneuver (ARM) are the most widely used PLV strategies and can reduce lung volume and pressure injuries, improve lung function, and reduce PPCs [15]. Intraoperative mechanical ventilation combined with PEEP has value in the prevention and treatment of postoperative atelectasis and other pulmonary complications, but the level of PEEP required among patients varies greatly. A single PEEP does not consider the individual differences between patients, which may affect the effect of PLV. If PEEP is too low, atelectasis cannot be prevented. If PEEP is too high, a lung pressure injury might occur and the thoracic pressure will be increased which together will be unfavorable with respect to blood reflux, thus affecting circulation stability [16, 17]. For patients undergoing craniotomy, PEEP may also affect intracranial pressure (ICP) and brain-blood reflux, affecting the surgical process and patient recovery.

Electrical impedance tomography (EIT) measures the changes in electrical impedance under different ventilation conditions and dynamically monitors the ventilation distribution in different lung regions in real time [18]. EIT has the outstanding characteristics of non-invasiveness, radiation-free, and portable, which makes EIT possible for patients with mechanical ventilation under general anesthesia to quickly obtain individualized PEEP.

At present, individualized PEEP is mainly used for the study of postoperative atelectasis and static compliance [19, 20]; the impact of individualized PEEP on the incidence and severity score of PPCs has not been reported. The use of PEEP during craniotomy may affect cerebral blood flow reflux, lead to brain tissue swelling, and affect the surgical procedure. After lesion clearance, it often takes some time from dura suturing to extubation. Individualized PEEP ventilation during this time can not only avoid the impact on surgery, but also may reduce the incidence of PPCs. The purpose of this study is to determine whether EIT-guided individualized PEEP during the time from dura suturing to extubation reduces the incidence of pulmonary complications within 1 week after craniotomy. Other outcomes include the severity of pulmonary complications within 1 week, the lung ultrasound score (LUS), regional brain oxygen saturation (rScO_2_) and PaO_2_/FiO_2_ (before anesthesia and 10 min, 24 h, and 72 h after extubation), and the postoperative ICU and hospital stay.

## Methods

### Study design

**Fig. 1:**
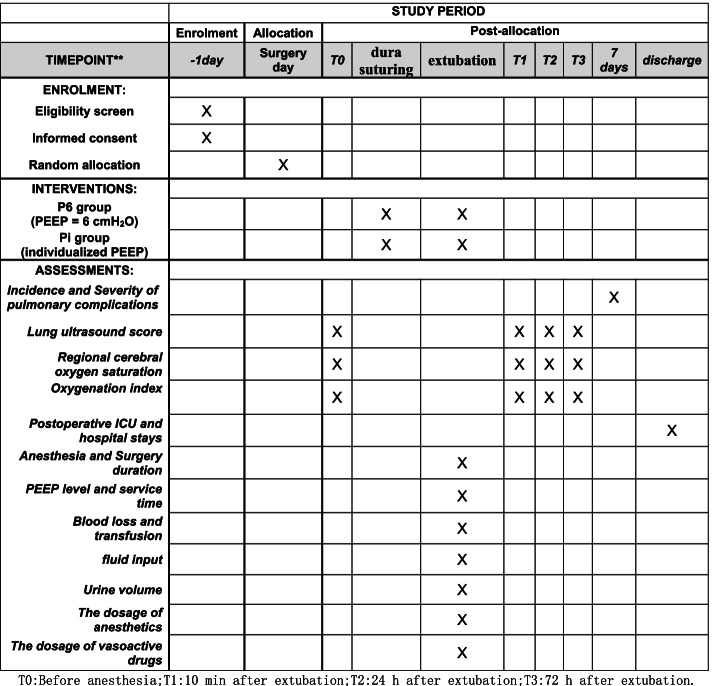
Schedule of enrollment, interventions, and assessments

This is a single-center, randomized controlled, single-blind trial that has been approved by the Ethics Committee of the Second Affiliated Hospital of Soochou University (no. JD-LK-2018-077-01) and registered in the Chinese Clinical Trial Registry (ChiCTR, no. CHiCTR2100051200). The framework of the study is to explore the superiority of individual PEEP compared with single PEEP. The schedule of enrollment, interventions, and assessments is shown in Fig. 1. The Standard Protocol Items: Recommendations for Interventional Trials (SPIRIT) checklist is provided as Additional file 1.

### Selection and withdrawal of patients

#### Recruitment

Patients admitted to the Neurosurgery Department of the Second Affiliated Hospital of Soochow University for supine surgery under general anesthesia will be screened and recruited during a routine preoperative evaluation. The basic patient information is shown in Table 1. Participants who meet the inclusion criteria will be required to sign an informed consent. The designated staff who performs the preoperative visit will be responsible for obtaining the informed consent.

**Table 1:** Baseline patient characteristics

Variables	P6 group	Pi group	*P* value
Gender (male/female)			
Age (years)			
Height (cm)			
Weight (kg)			
BMI (kg/m^2^)			
ASA classification (I–II)			
Smoking (cases): never/ever/within 1 month before surgery			

#### Inclusion criteria

The inclusion criteria will be as follows: (1) patients undergoing elective craniotomy in the supine position, (2) 18–65 years of age, (3) American Society of Anesthesiologists (ASA) classification I–II, (4) 18.5 kg/m^2^ < BMI < 28 kg/m^2^, and (5) agree to participate in this study and sign an informed consent for anesthesia.

#### Exclusion criteria

Patients will be excluded from the study if they have any of the following conditions: (1) decline to participate in the study; (2) chronic lung diseases such as asthma, COPD, restrictive diseases, pneumonia, atelectasis, pleural effusion within 1 month before surgery, and acute lung injury caused by various reasons; (3) history of lung surgery, (4) abnormal cardiac function, (5) intracranial hypertension; and (6) preoperative CT or MRI showing midline deviation and ventricular compression.

#### Randomization

All participants who meet the inclusion criteria will be randomly divided into two groups, P6 group (PEEP = 6 cmH_2_O) or Pi group (individualized PEEP) in a ratio of 1:1. Randomization will be achieved using a computer-generated randomization table. The designated staff will perform the allocation sequence and assign participants to the interventions. This research staff will implement the distribution sequence through sealed and opaque envelopes. Participants will not open the corresponding envelopes until they have completed the experiment. The patients, surgeons, and staff involved in data collection and follow-up visits will be blinded to the grouping; however, the anesthesiologist responsible for the implementation of anesthesia will be aware of the grouping.

### Methods

#### Related parameter setting during operation

Upon receiving the patients in the operating room, the following will be monitored: noninvasive blood pressure, electrocardiogram (ECG), and pulse blood oxygen saturation. Invasive arterial pressure will be monitored by radial artery catheterization under local anesthesia. After completing the spontaneous breathing data record, intravenous anesthesia induction will be administered and endotracheal intubation will be performed: fentanyl, 5 μg/kg; etomidate, 0.3 mg/kg; and rocuronium, 0.6 mg/kg. After confirming the correct placement of the endotracheal tube, the Primus anesthesia machine (Drager, Germany) will be connected to the tube and volume-controlled mechanical ventilation will be initiated with the following parameters: tidal volume, 7 mL/kg; inhalation-expiration ratio (I: E) = 1:2; and oxygen flow, 1 L/min. The respiratory rate will be adjusted, and the partial pressure of exhaled carbon dioxide (PetCO_2_) will be maintained at 30~35 mmHg. Sevoflurane (1%) combined with propofol and remifentanil will be used to maintain anesthesia, and the bispectral index (BIS) will be maintained at 45–55. During the surgical procedure, fentanyl will be intermittently injected to deepen anesthesia, and rocuronium will be intermittently injected to maintain the single muscle twitch stimulation (T1/T0) on the muscle relaxation monitor < 25%. The intraoperative fluid intake and urine volume will be monitored closely. The tidal volume and drug dosage will be calculated according to the estimated body weight. The formulae to calculate the estimated body weight [21] are as follows: female (kg) = [height (cm) − 152.4] × 0.91 + 45.5 and male (kg) = [height (cm) − 152.4] × 0.91 + 50. All patients will receive an intravenous drip of 20% mannitol (1 g/kg) before opening the dura, and the head will be kept high and feet low (15°) during anesthesia and surgery. The indications for extubation will be an awake and cooperating patient and muscle relaxation monitoring train-of-four (TOF) stimulation > 90% [22]. The invasive blood pressure of patients before anesthesia induction will be designated as the basic blood pressure. When the systolic blood pressure, diastolic blood pressure, and mean arterial pressure (MAP) are 20% lower than the basic blood pressure or the MAP is < 60 mmHg, 0.1 mg/kg of ephedrine will be injected intravenously to increase the blood pressure. Any treatment that affects the stability of vital signs and is adverse to the rehabilitation of the patients is prohibited.

**Fig. 2:**
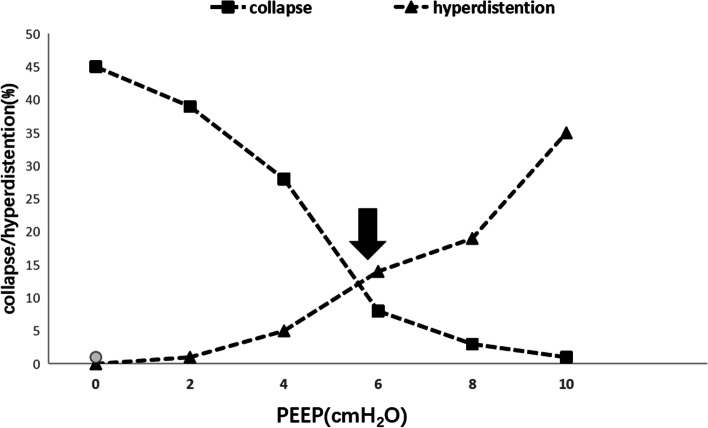
EIT-guided individualized PEEP titration

Based on the different options for lung protective ventilation during operation, the patients will be randomly allocated into two groups (P6 or Pi groups). In order to avoid the influence of PEEP on cerebral blood flow and intracranial pressure and thus affect the exposure of surgical field, PEEP (PEEP = 0 cmH_2_O) will not be used in both groups during the period from tracheal intubation to dura mater suturing. At the beginning of dura suturing, patients in both groups will undergo a lung recruitment maneuver. The airway pressure will be maintained at 30 cmH_2_O for 30 s [23], then the PEEP will be increased from 0 to 10 cmH_2_O in a 2-cmH_2_O gradient, and each gradient will be maintained for 1 min [24]. After the lung recruitment maneuver, 6 cmH_2_O PEEP will be used in the P6 group and EIT-guided individualized PEEP will be used in the Pi group until the endotracheal tube is removed. In the pre-test, the median of the best PEEP is 6 cmH_2_O, so we plan to compare individualized PEEP with a single PEEP ( PEEP = 6 cmH_2_O ). The EIT-guided individualized PEEP titration method is as follows [19]: all patients will wear an EIT (PV500, Dräger, Germany) electrode belt (including 16 electrodes) before anesthesia. During the increase in PEEP from 0 to 10 cmH_2_O, the data of pulmonary electrical impedance at each PEEP will be obtained. The EIT Data Analysis Tool (6.1) will be used to analyze the insufficient ventilation and excessive expansion areas at different PEEP and plot a graph (Fig. 2). EIT-guided individualized PEEP is considered as the nearest PEEP above the crossing of the curves representing overdistension and collapse.

#### Primary and secondary outcomes

The primary outcome of this study is the incidence of pulmonary complications within 1 week following surgery. According to the method adopted by Costa-Leme et al. [25], the modified PPC scoring table will be used as the standard to evaluate the incidence of PPCs after surgery. When a patient has any of the symptoms or signs in Table 2 (e.g., cough, atelectasis, dyspnea, bronchospasm, hypoxemia, hypercapnia, pleural effusion, pneumonia, pneumothorax, mechanical ventilation, endotracheal intubation, respiratory failure) or the score ≥ 1, it indicates that the patient has postoperative pulmonary complications: incidence of pulmonary complications (%) = number of cases with pulmonary complications/total number of patients in this group.

**Table 2:** Modified scoring standard of PPCs

Postoperative pulmonary complication score	
**Grade 1**	- Dry cough.
	- Micro-atelectasis: abnormal pulmonary symptoms or signs, body temperature excluding extrapulmonary causes > 37.5 °C; radiologic examination is normal.
	- Dyspnea, excluding extrapulmonary causes.
**Grade 2**	- Cough, expectoration, excluding extrapulmonary causes.
	-Bronchospasm: wheeze or the original wheeze requires changes in treatment.
	- Hypoxemia: SpO_2_ ≤ 90% when breathing air.
	- Atelectasis: radiologic evidence, with a body temperature > 37.5 °C or abnormal lung symptoms or signs.
	- Hypercapnia requiring treatment (PaCO_2_ > 50 mmHg).
**Grade 3**	- Pleural effusion requiring pleural puncture for drainage.
	- Pneumonia: radiologic evidence, accompanied by clinical symptoms (two of the following symptoms: leukocytosis or leukopenia, abnormal body temperature and purulent secretion, and pathologic evidence (Gram staining or bacterial culture) or the use of antibiotics need to be changed).
	- Pneumothorax.
	- Patients need non-invasive mechanical ventilation in all of the following situations: (a) blood oxygen saturation (SpO_2_) is < 92% during oxygen inhalation; (b) during oxygen inhalation, the oxygen flow needs to be > 5 L/min; and (c) respiratory rate ≥ 30 times/min.
	- Endotracheal intubation again after surgery, ventilator-dependent time (non-invasive or invasive mechanical ventilation) ≤ 48 h.
**Grade 4**	- Respiratory failure: the postoperative ventilator-dependent time exceeds 48 h or the ventilator-dependent time exceeds 48 h after re-intubation.
**Grade 5**	- Death caused by respiratory system failure.

^*^Only when two or more conditions occur at the same time, it is classified as grade 2

Bedside chest radiographs will be obtained for all patients at the first, third, fifth, and seventh postoperative days, in addition to those requested by the staff surgeons. They are independently analyzed by 2 pulmonary specialists, blinded to the grouping. Only concordant assessments (an abnormal opacity at the same location) will be integrated into clinical findings for the final score of pulmonary complications. The occurrence of pulmonary complications will be assessed daily, until the 7th day following surgery.

The secondary outcomes of this study are as follows:

1. Severity of pulmonary complications within 1 week: The severity of pulmonary complications will be assessed daily, until 1 week after surgery, using the worst score within a week for analysis. The modified PPC scoring table will be scored on an ordinal scale ranging from 0 to 5, in which grade 0 represents no pulmonary complications, grades 1–4 represent gradually deteriorating pulmonary complications, and grade 5 represents death of patients caused by respiratory diseases before discharge (Table 2).
2. LUS: Lung ultrasound examination will be performed using the 2−5-MHz convex array ultrasonic probe of Edge (Sonosite Corporation, USA) before anesthesia (T0), 10 min after extubation (T1), 24 h after extubation (T2), and 72 h after extubation (T3). Twelve lung regions will be examined [26]; the lung on each side is divided into two areas by the fourth intercostal space: the upper and lower lung areas, and the upper and lower lung areas of the right and left lungs are delineated by the parasternal, anterior axillary, posterior axillary, and paravertebral lines. Therefore, both lungs are divided into 12 areas according to body surface markers. According to the improved scoring table (Table 3), the highest LUS of each lung area is 3 points and the lowest LUS is 0 points. Therefore, the total score of LUS ranges from 0 to 36 points. The higher the LUS, the more severe the atelectasis in this area [27].
3. Regional cerebral oxygen saturation (rScO_2_): The 5100C brain and regional blood oxygen detection system (Covidien Corporation, USA) will be used to monitor rScO_2_ at T0, T1, T2, and T3. The mean value of the bilateral rScO_2_ will be considered as the rScO_2_. To ensure the accuracy of data, rScO_2_ will be collected 3 times, with an interval of 10 s each time, and the average value of the 3 values will be calculated.
4. Arterial oxygen partial pressure/inspired oxygen fraction (PaO_2_/FiO_2_): arterial blood will be collected at T0, T1, T2, and T3 for blood gas analysis to determine the PaO_2_/FiO_2_.
5. Postoperative ICU and hospital stays.
6. Other research indicators include the duration of surgery and anesthesia, level and duration of PEEP, blood loss volume, blood transfusion volume, fluid input and urine output during surgery, and dosage of anesthetics and ephedrine during surgery (Table 4).

**Table 3:** Scoring criteria for lung ultrasound images

Score	Ultrasonic image
0 point	Clear A lines and pleural sliding sign with or without 0–2 B lines
1 point	≥ 3 B lines or small subpleural consolidations separated by smooth pleural lines
2 points	Multiple merged B lines or small subpleural consolidations separated by thickened and irregular pleural lines
3 points	> 1 × 2 cm subpleural consolidations

**Table 4:** Intraoperative characteristics

Variables	P6 group	Pi group	***P*** value
**Meningioma/glioma/others (cases)**			
**Maximum diameter of tumor (mm)**			
**Anesthesia duration (min)**			
**Surgery duration (min)**			
**Bleeding volume (mL)**			
**Urine volume (mL)**			
**Sodium lactate ringer injection (mL)**			
**Hydroxyethyl starch injection (mL)**			
**Concentrated red blood cells (U)**			
**Fentanyl dosage (mg)**			
**Rocuronium dosage (mg)**			
**Ephedrine dosage (mg)**			
**PEEP level (cmH**_**2**_**O)**			
**PEEP service time (min)**			

#### Withdrawal of research

Because participation in the trial will be voluntary, the patients will have the right to withdraw their consent to participate in the study at any time and for any reason without any further treatment. In addition, if the researcher believes that participation is not in the patient’s best interests, the researcher has the right to terminate his/her participation at any time. The reasons and circumstances for disenrolling from the study will be recorded in the case report form.

#### Reporting of adverse events

All adverse events will be treated immediately and monitored until stabilization or resolution. The chief investigator will be responsible for reporting the event immediately to the Endpoint Adjudication Committee. The severity and causality of the adverse events will be assessed and recorded. Compensation will be provided in accordance with the relevant national regulations in the event of test-related damage.

#### Data acquisition and management

All personal information will be collected through the hospitalized medical records and be kept strictly confidential for research purposes only. Only the primary investigator and the designated researcher can obtain interim results and final test data. The data monitoring of this study will be performed independently by a Data Monitoring Committee (DMC) composed of specialists in anesthesiology, ethics, statistics, and methodology. The progress of the study will be evaluated, and the accuracy and integrity of data records will be verified through regular interviews. At the end of the study, the original data and results will be submitted to the Scientific Research Management Committee and disclosed to the public after the study results are published. The study results will be published in peer-reviewed journals.

#### Sample size estimation and interim analysis

The main outcome of this study is the incidence of pulmonary complications 1 week after surgery. In the pre-test (20 cases in each group), the incidence of pulmonary complications 1 week after surgery in the P6 and Pi groups was 24% and 13.5%, respectively. Using the PASS15.0 procedure with *α* = 0.05 and *β* = 0.2, the minimum sample size of each group was calculated to be 216 cases. Based on the shedding rate of 20%, the number of cases included in each group will be at least 216 ÷ (1–20%) = 270. A total of 540 patients will need to be enrolled in the two groups in this study. The first patient was enrolled on 1 September 2021. The expected duration of the study is 25 months.

There will be a formal interim analysis performed by the Data Monitoring Committee to evaluate the efficacy of the primary outcome once 50% of the planned patients have been randomized and initiated intervention and have been followed up until discharge. The *p* value for the analysis will be set at *p*<0.001 using the alpha-sparing technique (O'Brien-Fleming) for benefit or harm. Stoping rules for adverse events: we will consider patients withdrawal from the trial if the following conditions occur. (1) severe brain swelling during surgery; (2) persistent hypotension and circulatory instability. 

#### Statistics

Data will be collected in a standardized form and transmitted to the Data Monitoring Committee whenever the patient is discharged from the hospital. The completeness and the quality of the data will be checked by a data collector from the Data Monitoring Committee. Logical checks will be performed for missing data and to find inconsistencies. When necessary, the data collector will contact the investigator by phone to validate the data or reformat the data before entry into the database.

The SPSS 21.0 software package will be used for statistical analysis. The counting data will be expressed as the number of patients (percentage) and be analyzed using the chi-square (*χ*^2^) test or Fisher exact test. The normally distributed quantitative variables will be expressed as the mean ± standard deviation (SD). Two independent sample *t*-tests will be used for comparison between groups, and analysis of variance (ANOVA) will be used for comparison at different time points within the groups. Quantitative variables of the skewness distribution will be expressed as the median (interquartile range [IQR]) or mean (95% confidence interval [CI]), and the Mann-Whitney *U* test will be used for comparison. *P* < 0.05 will indicate that the difference is statistically significant. Intention-to-treat will be considered for the patients with an incomplete follow-up period. Missing values will be handled by the mixed model for repeated measurements.

#### Trial organization

The steering committee is composed of principal investigators who contributed to the design and approval of the final protocol (Additional file 2). The Executive Committee is composed of the main investigators and is responsible for the administration, trial, and data collection. The data management team is composed of external independent experts in anesthesiology, statistics, and imaging. It is responsible for checking the integrity and reliability of the data, and it will recommend the continuation or discontinuation of the trial according to the results of the interim analysis. The trial overseeing group is composed of a project manager and experts in methodology and ethics. It is responsible for monitoring recruitment rate, clinical intervention, and follow-up and arranging a research progress meeting with principal investigators every month.

## Discussion

This study is a single-center randomized controlled trial to determine if the pulmonary impedance-guided individualized PEEP during the period from dura suturing to extubation reduces the incidence and severity score of pulmonary complications within 1 week following a craniotomy compared with a single PEEP. Considering the effects of anesthesia duration, age, insulin-dependent diabetes mellitus, and chronic obstructive pulmonary disease on PPCs [28], all patients will have a BMI ranging from 18.5 to 28 kg/m^2^, are 18~65 years of age without diabetes, and have co-existing lung diseases to avoid the effect of these factors on outcome. PPCs are defined as clinically relevant and identifiable lung changes that adversely affect the prognosis of patients. Because this definition lacks accuracy in the diagnosis of PPCs, the reported incidence of PPCs varies considerably [29]. Some researchers regard the comprehensive results of multiple lung diseases as lung complications, and others regard a specific lung disease or respiratory condition requiring special treatment postoperatively as lung complications [30, 31]. In this study, the improved PPCs score table will be used as the standard to evaluate the incidence of PPCs after surgery. The PPCs will be evaluated from multiple perspectives, such as symptoms, signs, blood gas analysis, and imaging changes, so that the evaluation is more systematic and comprehensive, and the severity of pulmonary complications can also be graded.

PEEP has been used clinically for more than 30 years because PEEP can prevent alveolar collapse, improve functional residual capacity, and improve lung dispersion and compliance [32–34]. Sreejit et al. [35] reported that the application of PEEP during mechanical ventilation under general anesthesia can effectively prevent alveolar collapse and expand the collapsed alveoli, thus improving hypoxemia; however, due to individual differences between patients, standardized and fixed PEEP cannot be applied to all patients. There is still controversy regarding the optimal level of PEEP during surgery [36, 37]. Franchineau et al. [38] conducted a study on individualized PEEP on 15 patients requiring extracorporeal membrane oxygenation, and the results showed that PEEP varies greatly among different patients, which suggested the necessity of individualized PEEP.

EIT-guided PEEP titration is a PEEP titration strategy that has attracted much attention in recent years. EIT images show the percentage of collapse and overexpanded areas corresponding to different PEEP levels in the total ventilation area, from which a curve of the percentage of collapse and overexpanded area can be drawn. The PEEP corresponding to the intersection of the two curves facilitates alveolar re-expansion as much as possible, while minimizing overexpansion, and thus, it is determined as the best PEEP. In the pre-experiments, the median PEEP obtained by EIT was 6 cmH_2_O. Therefore, we compare Pi with P6.

Considering that PEEP may increase thoracic pressure, affect hemodynamic stability, further increase intracranial pressure, affect cerebral perfusion, and have an impact on rScO_2_, we have chosen to apply PEEP for mechanical ventilation from the beginning of dura suturing to the end of the surgical procedure and before extubation to avoid its impact on the key stage of surgery. The doses of vasoactive drugs, PaO_2_/FiO_2_, and regional brain oxygen saturation will be used as observation indices to clarify the effectiveness and safety of individualized PEEP. Studies have shown that in patients with severe and acute brain injuries, the application of PEEP has no effect on intracranial pressure (ICP) or cerebral perfusion pressure (CPP) for those without severe lung injuries [39]. The increase in intracranial pressure is modest over the range of applied PEEP values (0–25 cmH_2_O), suggesting that PEEP may be safely applied to most mechanically ventilated patients with severe brain injuries. Therefore, we believe that it is relatively safe to titrate individualized PEEP in the range of 0–10 cmH_2_O. The purpose of this study is to explore the application value of pulmonary electrical impedance-guided individualized PEEP in reducing pulmonary complications within 1 week after a craniotomy. If it can be demonstrated that individualized PEEP reduces the incidence and severity score of pulmonary complications within 1 week after a craniotomy, showing a statistical difference or clinical significance, the study will promote the wide application of individualized PEEP in clinical anesthesia, further improve the prognosis of neurosurgical patients, and reduce the cost of hospitalization.

### Trial status

The study was registered on the registry website http://chictr.org.cn/ with registration number CHiCTR2100051200 on 15 September 2021. The protocol version is 1.0, dated 20 September 2021. The study began on 1 September 2021, and the planned completion date will be 30 September 2023. The trial status is currently recruiting. Recruitment began on 1 September 2021, and the planned recruitment completion date will be June 2023.

## Supplementary Information

**Additional file 1.** SPIRIT Checklist.

**Additional file 2.** Ethical Review Approval.

## Data Availability

The full protocol and datasets analyzed during the current study are available from the corresponding author upon reasonable request.

## Abbreviations

EIT: Electrical impedance tomography
PEEP: Positive end-expiratory pressure
LUS: Lung ultrasound score
PPCs: Postoperative pulmonary complications
PLV: Protective lung ventilation
ARM: Alveolar recruitment maneuver
ICP: Intracranial pressure
rSO_2_: Regional cerebral oxygen saturation
PaO_2_: Partial pressure of oxygen
FiO_2_: Fraction of inspiration oxygen
BMI: Body mass index
ECG: Electrocardiogram
ETCO_2_: End-expiratory carbon dioxide
BIS: Bispectral index
MAP: Mean arterial pressure
PaCO_2_: Partial pressure of carbon dioxide
SpO_2_: Pulse oxygen saturation
ICP: Intracranial pressure
CPP: Cerebral perfusion pressure
VT: Tidal volume

## References

[1] Blum JM, Stentz MJ, Dechert R (2013). Preoperative and intraoperative predictors of postoperative acute respiratory distress syndrome in a general surgical population. Anesthesiology..

[2] Haines KJ, Skinner EH, Berney S (2013). Association of postoperative pulmonary complications with delayed mobilisation following major abdominal surgery: an observational cohort study. Physiotherapy..

[3] Toledo C, Nácul FE, Knibel MF (2017). Pulmonary complications after non-cardiac surgeries: temporal patterns and risk factors. Anaesthesiol Intensive Ther.

[4] Miskovic A, Lumb AB (2017). Postoperative pulmonary complications. Br J Anaesth.

[5] Smetana GW, Lawrence VA, Cornell JE (2006). Preoperative pulmonary risk stratification for noncardiothoracic surgery: systematic review for the American College of Physicians. Ann Intern Med.

[6] Qaseem A, Snow V, Fitterman N (2006). Risk assessment for and strategies to reduce perioperative pulmonary complications for patients undergoing noncardiothoracic surgery: a guideline from the American College of Physicians. Ann Intern Med.

[7] Arozullah AM, Khuri SF, Henderson WG (2001). Development and validation of a multifactorial risk index for predicting postoperative pneumonia after major noncardiac surgery. Ann Intern Med.

[8] Sogame LC, Vidotto MC, Jardim JR (2008). Incidence and risk factors for postoperative pulmonary complications in elective intracranial surgery. J Neurosurg.

[9] Flexman AM, Merriman B, Griesdale DE (2014). Infratentorial neurosurgery is an independent risk factor for respiratory failure and death in patients undergoing intracranial tumor resection. J Neurosurg Anesthesiol.

[10] Fernandez-Bustamante A, Frendl G, Sprung J (2017). Postoperative pulmonary complications, early mortality, and hospital stay following noncardiothoracic surgery: a multicenter study by the Perioperative Research Network Investigators. JAMA Surg.

[11] Canet J, Gallart L, Gomar C (2010). Prediction of postoperative pulmonary complications in a population-based surgical cohort. Anesthesiology..

[12] Sabaté S, Mazo V, Canet J (2014). Predicting postoperative pulmonary complications: implications for outcomes and costs. Curr Opin Anaesthesiol.

[13] Damian D, Esquenazi J, Duvvuri U (2016). Incidence, outcome, and risk factors for postoperative pulmonary complications in head and neck cancer surgery patients with free flap reconstructions. J Clin Anesth.

[14] Mercado P, Maizel J, Kontar L (2018). Moderate and severe acute respiratory distress syndrome: hemodynamic and cardiac effects of an open lung strategy with recruitment maneuver analyzed using echocardiography. Crit Care Med.

[15] Hew M, Tay TR (2016). The efficacy of bedside chest ultrasound: from accuracy to outcomes. Eur Respir Rev.

[16] Mazzinari G, Diaz-Cambronero O, Alonso-Iñigo JM (2020). Intraabdominal Pressure targeted positive end-expiratory pressure during laparoscopic surgery: an open-label, nonrandomized, crossover, clinical trial. Anesthesiology..

[17] Spadaro S, Mauri T, Böhm SH (2018). Variation of poorly ventilated lung units (silent spaces) measured by electrical impedance tomography to dynamically assess recruitment. Crit Care.

[18] Bodenstein M, Boehme S, Bierschock S (2014). Determination of respiratory gas flow by electrical impedance tomography in an animal model of mechanical ventilation. BMC Pulm Med.

[19] Pereira SM, Tucci MR, Morais CCA (2018). Individual positive end-expiratory pressure settings optimize intraoperative mechanical ventilation and reduce postoperative atelectasis. Anesthesiology..

[20] Fernandez-Bustamante A, Sprung J, Parker RA (2020). Individualized PEEP to optimise respiratory mechanics during abdominal surgery: a pilot randomised controlled trial. Br J Anaesth.

[21] Treschan TA, Kaisers W, Schaefer MS (2012). Ventilation with low tidal volumes during upper abdominal surgery does not improve postoperative lung function. Br J Anaesth.

[22] Park M, Ahn HJ, Kim JA (2019). Driving pressure during thoracic surgery: a randomized clinical trial. Anesthesiology..

[23] Fogagnolo A, Montanaro F, Al-Husinat L (2021). Management of intraoperative mechanical ventilation to prevent postoperative complications after general anesthesia: a narrative review. J Clin Med.

[24] Ferrando C, Tusman G, Suarez-Sipmann F (2018). Individualized lung recruitment maneuver guided by pulse-oximetry in anesthetized patients undergoing laparoscopy: a feasibility study. Acta Anaesthesiol Scand.

[25] Costa Leme A, Hajjar LA, Volpe MS (2017). Effect of intensive vs moderate alveolar recruitment strategies added to lung-protective ventilation on postoperative pulmonary complications: a randomized clinical trial. JAMA..

[26] Xia J, Qian CY, Yang L (2019). Influence of lung aeration on diaphragmatic contractility during a spontaneous breathing trial: an ultrasound study. J Intensive Care.

[27] Monastesse A, Girard F, Massicotte N (2017). Lung ultrasonography for the assessment of perioperative atelectasis: a pilot feasibility study. Anesth Analg.

[28] Longo M, Agarwal V (2019). Postoperative pulmonary complications following brain tumor resection: a national database analysis. World Neurosurg.

[29] Fisher BW, Majumdar SR, McAlister FA (2002). Predicting pulmonary complications after nonthoracic surgery: a systematic review of blinded studies. Am J Med.

[30] Watkins TR, Nathens AB, Cooke CR (2012). Acute respiratory distress syndrome after trauma: development and validation of a predictive model. Crit Care Med.

[31] Hua M, Brady JE, Li G (2012). A scoring system to predict unplanned intubation in patients having undergone major surgical procedures. Anesth Analg.

[32] Hooda B, Chouhan RS, Rath GP (2019). Incidence and predictors of postoperative pulmonary complications in patients undergoing craniotomy and excision of posterior fossa tumor. J Anaesthesiol Clin Pharmacol.

[33] Zhang L, Xiong W, Peng Y, Zhang W, Han R (2018). The effect of an intraoperative, lung-protective ventilation strategy in neurosurgical patients undergoing craniotomy: study protocol for a randomized controlled trial. Trials..

[34] Manzano F, Fernández-Mondéjar E, Colmenero M (2008). Positive-end expiratory pressure reduces incidence of ventilator-associated pneumonia in nonhypoxemic patients. Crit Care Med.

[35] Sreejit MS, Ramkumar V (2015). Effect of positive airway pressure during pre-oxygenation and induction of anaesthesia upon safe duration of apnoea. Indian J Anaesth.

[36] Lima WA, Campelo AR, Gomes RL (2011). The impact of positive end-expiratory pressure on cerebral perfusion pressure in adult patients with hemorrhagic stroke. Rev Bras Ter Intensiva.

[37] Shapiro HM, Marshall LF (1978). Intracranial pressure responses to PEEP in head-injured patients. J Trauma.

[38] Franchineau G, Brechot N, Lebreton G (2017). Bedside contribution of electrical impedance tomography to setting positive end-expiratory pressure for extracorporeal membrane oxygenation-treated patients with severe acute respiratory distress syndrome. Am J Respir Crit Care Med.

[39] Boone MD, Jinadasa SP, Mueller A (2017). The effect of positive end-expiratory pressure on intracranial pressure and cerebral hemodynamics. Neurocrit Care.

