# Supplementary material for: Effect of electrical impedance-guided PEEP in reducing pulmonary complications after craniotomy: study protocol for a randomized controlled trial
Source: Trials. 2022 Oct 1;23:837. doi: 10.1186/s13063-022-06751-6 (PMC9526950; doi:10.1186/s13063-022-06751-6)

**Ethical Review Approval**

| Approval ID | JD-LK-2018-07701 | | |
| --- | --- | --- | --- |
| Project name | To study the strategies of mechanical ventilation in patients undergoing neurosurgery by using electrical impedance tomography and pulmonary ultrasound. | | |
| Project source | □Drug clinical trials □Medical instruments clinical trials □Diagnostic reagents 🗷Research □New technology | | |
| Institution | Second Affiliated Hospital of Soochow University | | |
| [Department](javascript:;) | [Anesthesiology](javascript:;) [department](javascript:;) | Principal investigator | Hairui Liu |
| Category [of](javascript:;) [review](javascript:;) | 🗷 Initial review □Review(after initial review) □Amendment review | | |
| [Method](javascript:;) [of](javascript:;) [review](javascript:;) | 🗷 Quick review Chief judge: Haihua Shan, Yaping Yang  □Meeting review □Emergency meeting review | | |
| Documents [of](javascript:;) [review](javascript:;) | See attachment | | |
| Review comments of the ethics committee | | | |
| Upon review by ethics committee, the study was approved to carry out according to the documents reviewed.  Comments and Suggestions: 🗷No □Yes  The frequency of regular follow-up review in this study: □3 months □6 months 🗷123 months □Others | | | |
| Signature of chairman: Date： .    Ethics Committee of the Second Affiliated Hospital of Soochow University (seal) | | | |
| Disclaimer :(please read carefully))  1. The composition and working procedures of the ethics committee are in accordance with ICH-GCP, GCP norms and relevant national laws and regulations.  2. The investigator is requested to conduct the clinical study in accordance with the GCP principles and the protocol approved by the ethics committee to protect the subjects' health and rights.  3. If the main investigator is changed, or the clinical study protocol informed consent and other materials are modified during the study, please submit the application for amendment review and cannot be implemented without approval.  4. In case of serious adverse events, please timely submit serious adverse events report.  5. Whether the study starts or not, please follow up the review frequency regularly according to the regulations. Submit the research progress report one month in advance. The ethics committee has the right to change the review frequency regularly according to the progress and development of the study.  6. Please submit a report of the protocol violation, in case of serious protocol violation and continuous protocol violation, or any violation of the principles of the GCP that may adversely affect the rights and interests of the subjects, the health of the subjects or the scientific nature of the study.  7. If the study is suspended or terminated in advance, please timely submit the suspension and termination report. To complete the clinical study, please submit the study completion report in time.  8. This approval is valid for 3 years, and it will be invalid if overdue. If extension is needed, please apply to the ethics committee at least 1 month before the expiration of the approval.  9. If approval from the Human Genetic Resources Management Office is required according to the national regulations, please apply for it by yourself. Approval is obtained before implementation. | | | |
| The contact: Wenyan Hua, Qianqian Huang Tel: 0512-67783682  Address: Room 1608, Building 1, Second Affiliated Hospital of Soochow University, 1055 Sanxiang Road, Suzhou. Zip code: 215004 | | | |


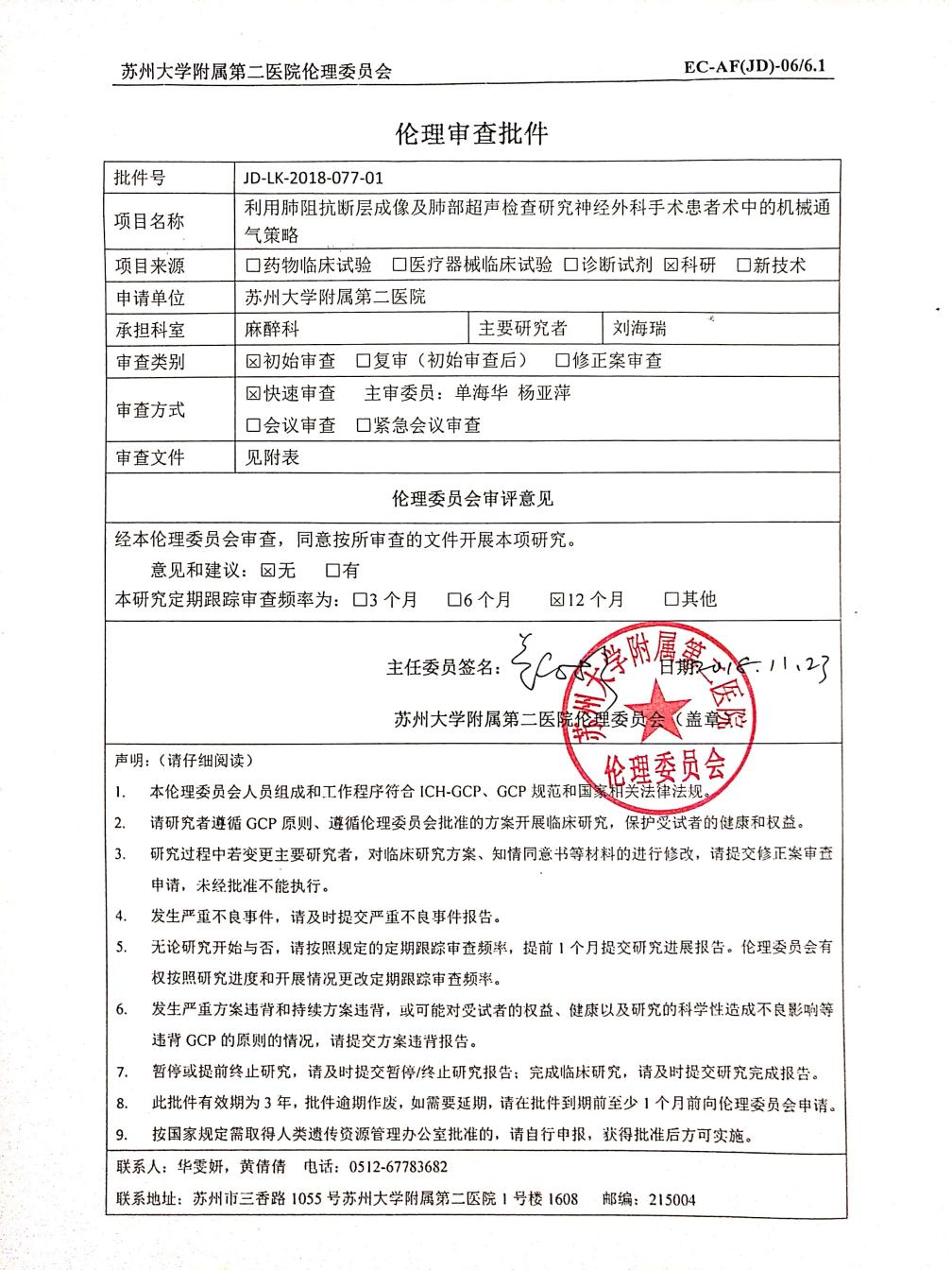

Supplement: Supplementary file 2 — Additional file 2. Ethical Review Approval. [file 13063_2022_6751_MOESM2_ESM.docx]
